# Supplementary material for: Epigenetic therapy to enhance therapeutic effects of PD-1 inhibition in therapy-resistant melanoma
Source: Melanoma Res. 2021 Nov 10;32(4):241–8. doi: 10.1097/CMR.0000000000000791 (PMC9245557; doi:10.1097/CMR.0000000000000791)
Supplement: Supplementary file 1 [file mr-32-241-s001.pdf]

Supplementary Fig. 1. Gating strategy for flow cytometry analyses. (a) A gating strategy for excluding debris and choosing tumor cells based on high forward scatter (FSC) was employed and used for estimating levels of HLA-A, -B, and -C, PD-L1 and HLA-DP, -DQ and -DR in different cell lines. Experiments from entinostat-treated UM22 cells are shown as representative examples. (b) Granzyme B and cleaved caspase 3 measurements in cell lines co-cultured with MART-1-reactive TILs. Experiments from MP41 are shown as representative examples. (c) Within the in vivo B16-F10 tumor suspension, leukocytes were identified by a low side scatter (SSC) and low forward scatter (FSC) with gates for estimating levels of live CD3+ cells for CD4+ and CD8+ TILs, as well as (d) gates for estimating levels of live CD45 cells for CD11b+, Ly6c+, Ly6g+, CD206+ myeloid infiltrating cells.

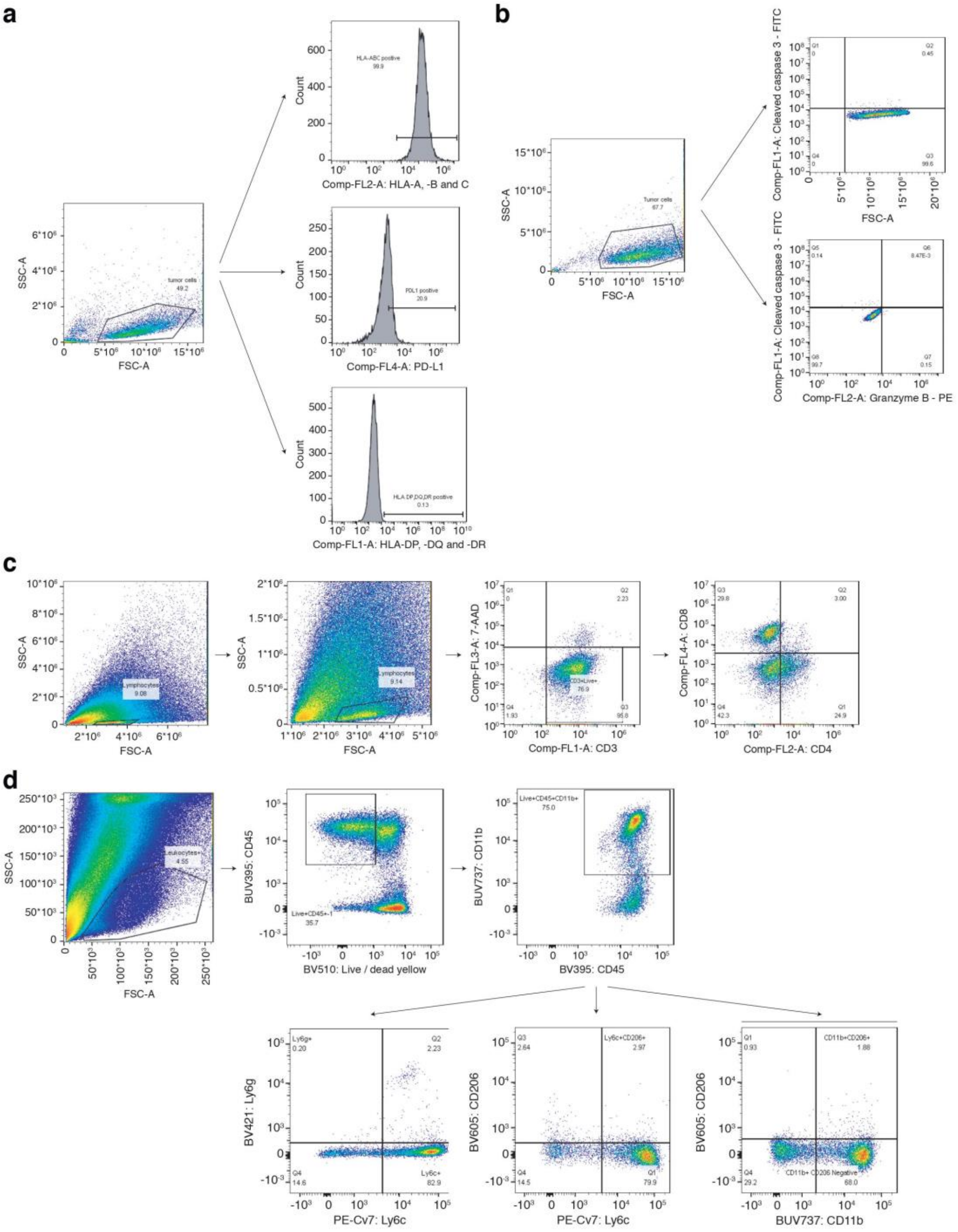
a
SSC-A
FSC-A
tumor cells
49.2
Count
HLA-ABC positive
99.9
Comp-FL2-A: HLA-A, -B and C
PDL1 positive
20.9
Comp-FL4-A: PD-L1
HLA DP,DQ,DR positive
0.13
Comp-FL1-A: HLA-DP, -DQ and -DR
b
Tumor cells
67.7
Comp-FL1-A: Cleaved caspase 3 - FITC
Comp-FL2-A: Granzyme B - PE
c
Lymphocytes
9.08
9.14
Comp-FL3-A: 7-AAD
CD3+Live+
76.9
Comp-FL1-A: CD3
Comp-FL4-A: CD8
Comp-FL2-A: CD4
d
Leukocytes+
4.65
BUV395: CD45
Live+CD45+-1
35.7
BV510: Live / dead yellow
BUV737: CD11b
Live+CD45+CD11b+
75.0
BV395: CD45
BV421: Ly6g
Ly6g+
0.20
Ly6c+
82.9
PE-Cv7: Ly6c
BV605: CD206
Ly6c+CD206+
2.97
CD11b+CD206+
1.88
CD11b+ CD206 Negative
68.0
BUV737: CD11b

Supplementary Fig. 2. Entinostat increases HLA expression in human UM cell lines and mouse B16-F10 melanomas. (a) HLA class 2 expression as assessed by flow cytometry in human UM cell lines 92-1, MP41 and UM22 treated with DMSO or entinostat. The experiment was repeated twice with n = 3 biological replicates per cell line and condition, except in the case of UM22, where n = 5 and n = 1 replicates were used for the first and second experiments, respectively (excluded from statistical tests due to nearly absent expression in all cases). Significance was assessed by t-tests and adjusted p-values <0.05 (Benjamini-Hochberg correction) were considered statistically significant, as indicated by asterisks. (b) Immune-associated gene expression levels inferred from RNA sequencing data after entinostat treatment, relative to DMSO controls, as shown in Figure 1c. (c) Flow cytometry analysis of parental B16-F10 cells and CRISPR/Cas9-generated PD-L1 knockout B16-F10 cells after treatment with entinostat for 24 h.

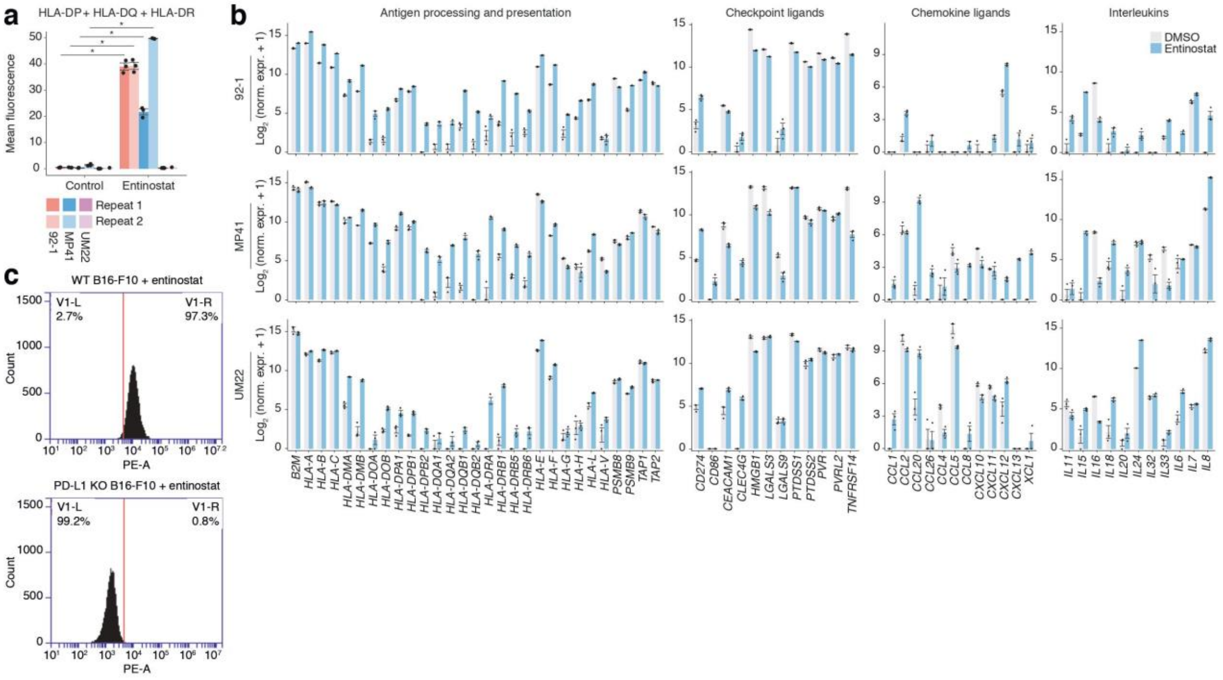
a
HLA-DP+ HLA-DQ + HLA-DR
Mean fluorescence
Control
Entinostat
Repeat 1
Repeat 2
92-1
MP41
UM22
b
Antigen processing and presentation
Checkpoint ligands
Chemokine ligands
Interleukins
DMSO
Entinostat
92-1
MP41
UM22
Log2 (norm. expr. + 1)
B2M HLA-A HLA-B HLA-C HLA-DMA HLA-DMB HLA-DOA HLA-DOB HLA-DPA1 HLA-DPB1 HLA-DPB2 HLA-DQA1 HLA-DQA2 HLA-DQB1 HLA-DQB2 HLA-DRA HLA-DRB1 HLA-DRB5 HLA-DRB6 HLA-E HLA-F HLA-G HLA-H HLA-L HLA-V PSMB8 PSMB9 TAP1 TAP2
CD274 CD86 CEACAM1 CLEC4G HMGB1 LGALS3 LGALS9 PTDSS1 PTDSS2 PVR PVRL2 TNFRSF14
CCL1 CCL2 CCL20 CCL26 CCL4 CCL5 CCL8 CXCL10 CXCL11 CXCL12 CXCL13 XCL1
IL11 IL15 IL16 IL18 IL20 IL24 IL32 IL33 IL6 IL7 IL8
c
WT B16-F10 + entinostat
V1-L
2.7%
V1-R
97.3%
Count
PE-A
PD-L1 KO B16-F10 + entinostat
V1-L
99.2%
V1-R
0.8%
Count
PE-A
